# Viral Determinants of FeLV Infection and Pathogenesis: Lessons Learned from Analysis of a Natural Cohort

**DOI:** 10.3390/v3091681

**Published:** 2011-09-09

**Authors:** Lisa L. Bolin, Laura S. Levy

**Affiliations:** 1 Department of Microbiology and Immunology, School of Medicine, Tulane University, 1430 Tulane Avenue SL-38, New Orleans, LA 70112, USA; E-Mail: lbolin@tulane.edu; 2 Tulane Cancer Center, School of Medicine, Tulane University, 1430 Tulane Avenue SL-38, New Orleans, LA 70112, USA

**Keywords:** feline leukemia virus, lymphoma, pathogenesis, oncogenesis, long terminal repeat, surface glycoprotein

## Abstract

Detailed analysis has been performed over many years of a geographic and temporal cohort of cats naturally infected with feline leukemia virus (FeLV). Molecular analysis of FeLV present in the diseased tissues and application of those viruses to experimental systems has revealed unique isolates with distinctive disease potential, previously uncharacterized virus-receptor interactions, information about the role of recombinant viruses in disease induction, and novel viral and cellular oncogenes implicated in pathogenesis, among other findings. The studies have contributed to an understanding of the selective forces that lead to predominance of distinctive FeLV isolates and disease outcomes in a natural population.

## 1. Introduction

**Figure 1.**
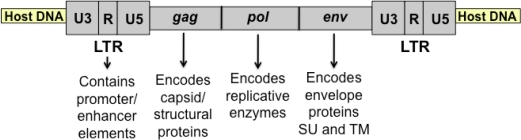
Diagrammatic representation of integrated feline leukemia virus (FeLV) proviral DNA. After reverse transcription of viral RNA, the double-stranded DNA provirus is integrated into the host genome as shown. Indicated are the viral genes (*gag*, *pol*, and *env*) and long terminal repeat (LTR).

Feline leukemia virus (FeLV) is a horizontally-transmissible, naturally occurring retrovirus of the domestic cat, associated with proliferative, degenerative and malignant diseases of myeloid, erythroid, and lymphoid origin in the natural host. This complex disease spectrum is thought to reflect a prolonged interaction *in vivo* between two genetically heterogeneous populations, *i.e.*, FeLV itself and its naturally outbreeding host. As a simple gammaretrovirus, FeLV encodes only those genes required for its structure and replication and carries no genetic information to which its disease potential can be directly attributed (Figure 1). In contrast to the relative simplicity of its genome, FeLV occurs in nature not as a single genetic species but as a complex family of closely related viruses. Genetic variation is generated during FeLV replication *in vivo* through error-prone reverse transcription and by recombination with endogenous FeLV-related sequences in the cat genome. The consequence of this variation is a genetically diverse virus population that is continuously shaped by selective pressures *in vivo* and from which variants arise as predominant species [1–3]. The nature of these variants, their relationship to disease outcome, particularly malignant disease, and the selective mechanisms leading to their predominance have been topics of research focus in our laboratory for many years. The malignant disease most frequently associated with FeLV infection is a lymphoma characterized by the presence of discrete tumors and diffuse infiltration of organs by lymphoid tumor cells. Three forms of lymphoma have been described clinically in the FeLV-infected cat: (1) thymic, a rapidly progressive tumor of the anterior mediastinum comprised of T-lineage cells at varying stages of developmental maturity, (2) alimentary, in which the tumor involves the gastrointestinal tract, and (3) multicentric, in which the tumor involves many organs but typically excludes the thymus [4]. Our research goal has been to dissect the complex, multistep cascade of events that leads to lymphoma in FeLV-infected cats, and thereby identify the role of viral determinants.

**Figure 2.**
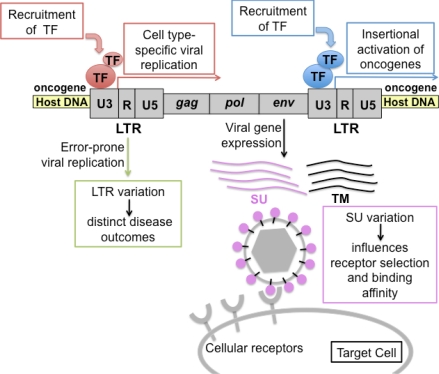
Schematic representation of the pathogenic determinants of FeLV. Transcriptional promoter and enhancer elements in the U3 region of the viral LTR bind transcription factors (TF) to drive expression of viral genes. Host genes near the site of integration may be similarly affected. If the adjacent host gene is an oncogene, activation of its expression can lead to malignant change. LTR variants, produced by error-prone replication, are associated with distinct disease outcomes through the engagement of distinct sets of generalized or cell type-specific transcription factors. Variation in the surface glycoprotein (SU) affects receptor selection and binding affinity, and can thereby influence the rate of virus spread, tissue tropism and disease spectrum.

Insight into the interplay between FeLV and its host was afforded by the extraordinary opportunity to examine diseased tissues from a cohort of naturally infected cats, the generous gift of Drs. Murray Gardner and James W. Casey. A rare trove of clinical material, the cohort included tissues from 66 animals collected essentially by a single veterinary practice in Pasadena, California over a period of six years. Thus, the cohort represented a geographical and temporal cluster presumably exposed to a similar spectrum of horizontally transmissible FeLV. The cohort included twelve cases of multicentric lymphoma, four cases of thymic lymphoma, two cases of myeloproliferative disease, one case of mast cell leukemia, two cases of anemia, and two asymptomatic cases in FeLV-infected, healthy animals. The thymic lymphomas were demonstrated to be of T-cell origin as evidenced by clonal rearrangement of the T-cell receptor beta (TCRβ) locus, but the cell type of origin of the multicentric lymphomas could not be clearly determined. As both TCRβ and immunoglobulin heavy chain (IgH) loci were observed in germline configuration in those tumors [5], we have referred to them as non-B-cell non-T-cell multicentric lymphomas. The designation is tentative, in that no intact tissues were available for immunohistochemical or other analyses. Our studies of lymphomas and other diseased tissues in the cohort have shown that FeLV-induced malignancy is a multistep process that involves determinants encoded both by the virus and the host. The malignant potential of FeLV, like the closely related murine leukemia viruses [6], was shown to depend on at least three genetic determinants: (1) transcriptional regulatory sequences in the virus long terminal repeat (LTR), (2) potential influences on target cell tropism and spread *in vivo* by variation in the envelope surface glycoprotein (SU), and (3) the activation of cellular oncogenes, typically by the adjacent integration of a transcriptionally active provirus (Figure 2). Described below are investigations of the role(s) of these determinants in the pathogenesis of FeLV-positive thymic and multicentric lymphomas in the natural cohort. The results shed light on selective pressures operative in natural FeLV infection that led to the predominance of viral variants, many of which have significant consequences for infection and disease outcome. The results have further associated a clearly distinguishable set of genetic events with lymphomas of each type.

## 2. The FeLV Long Terminal Repeat (LTR) as a Determinant of Pathogenesis in the Cohort

**Figure 3.**
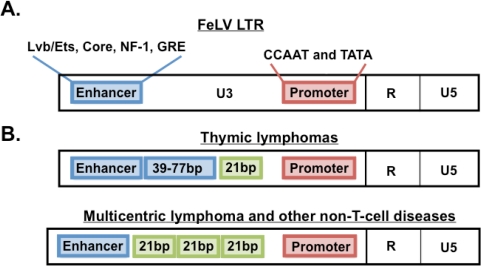
Structure of FeLV LTR and its natural variants. (**A**) FeLV LTR structure as observed typically in asymptomatic infections in nature. The LTR contains a single transcriptional enhancer and promoter element as shown. Indicated are transcription factors that bind their cognate sites within those elements. (**B**) FeLV LTR structure observed in thymic lymphomas or in non-T-cell diseases from the natural cohort. In thymic lymphomas, LTRs frequently contain a duplicated enhancer with repeat lengths varying from 39–77 base pairs (bp). In non-T cell diseases, LTRs frequently contain a single copy of enhancer followed downstream by a 21-bp sequence triplicated in tandem.

Gene expression in retroviruses is directed by the LTR, a structure generated at the termini of the proviral genome by reverse transcription. The LTR is a modular structure consisting of a DNA form of the U3, R, and U5 regions of viral RNA (Figure 3A). The U3 is particularly relevant for the regulation of gene expression as it contains the transcriptional promoter and potent enhancer sequences. These regulatory elements can act both on adjacent viral genes and on cellular genes near the site of proviral integration, or indeed across significant sequence distance. As such, the LTR of FeLV is capable of directing a high level of viral gene expression and can also direct expression of host genes. When such host genes have oncogenic potential, the LTR-mediated activation plays a principal role in the malignant process [6].

The FeLV LTR represents a region of remarkable genetic variation among natural isolates, and LTR variants have been linked to particular disease outcome [7–11]. LTR sequence variation and function were examined in diseased tissues of 21 cats in our collection (Figure 3B). In thymic lymphomas in the cohort, LTRs were found uniformly to contain duplications of enhancer sequences with repeat lengths varying from 39 to 77 base pairs (bp). Termini of the enhancer repeat units varied among isolates, although the LVb/Ets and Core binding sites were uniformly conserved within the repeat units regardless of length. Unexpectedly, functional assays using luciferase reporter genes driven by the LTRs demonstrated that the enhancer duplication offered little transcriptional advantage, and that the more complete repeat unit conferred no advantage over a shorter one [12]. Working together with Dr. Jack Lenz, one of the thymic lymphoma-derived FeLV LTRs was further examined by substitution into the potent T-lymphomagenic murine leukemia virus, SL3-3 MuLV, to generate a murine-feline recombinant virus. When inoculated into mice, the recombinant virus induced T-cell lymphoma nearly as quickly as the parent murine leukemia virus. We interpreted this finding as confirmation that the thymic lymphoma-derived FeLV LTR contains a potent genetic determinant of T-cell lymphomagenesis. We infer that the LTR is presumably adapted, through the conservation of key binding sites, to be recognized by transcription factors present in T cells of cats in the relevant target tissues for malignant change [13].

In non-T-cell diseases in the cohort, by contrast, the predominant LTR structure contained a unique, previously unidentified sequence motif. Originally described from a non-T-cell, non-B-cell multicentric lymphoma, the LTR was shown to contain a single copy of the canonical enhancer followed 25-bp downstream by a 21-bp sequence triplicated in tandem [14]. PCR amplification of LTRs from all non-T-cell diseases in the cohort demonstrated the triplication-containing LTR in 7 of 12 multicentric lymphomas and in all cases of myeloproliferative disease and anemia. Unlike the enhancer duplications in LTRs from thymic lymphomas, whose repeat length and termini were variable, the sequence and position of the 21-bp repeat element was precisely conserved in the LTRs from all tissues in which it was identified. The FeLV isolate bearing the triplication-containing LTR was designated FeLV-945 [5,12]. Functional analysis using reporter gene constructs demonstrated that the 21-bp triplication provides transcriptional enhancer function to the FeLV-945 LTR, and that it acts preferentially in a cell-type-specific manner [15]. These results predicted that the FeLV-945 LTR would confer a replicative advantage on the virus that contains it, and that such an advantage might account for the precise conservation and selection of the unique LTR sequence in the cohort. A test of this prediction was performed using recombinant, infectious FeLVs isogenic other than the LTR structure. These studies demonstrated that the FeLV-945 LTR confers a significant replicative advantage to the virus, especially in multipotential hematopoietic cells and in feline T-cells [16]. Others have determined that a mutation which affords as little as 1% replication advantage will represent 50% of the virus population within 400 replication cycles, assuming a mutation rate of 10^−4^ [17]. Thus, a replicative advantage conferred by the FeLV-945 LTR may indeed have contributed to predominance of the unusual isolate in the cohort. In fact, LTRs from non-T-cell diseases in the cohort demonstrated the 21-bp repeat element in 1, 2, 3, or 4 copies, but the triplicated form as represented in FeLV-945 was observed to confer the optimal replicative advantage, perhaps explaining its predominance [12].

Two possible mechanisms of action have been examined to explain the function of the 21-bp triplication in the context of the FeLV-945 LTR. First, we considered that the 21-bp triplication might function to maintain the appropriate spacing in the LTR between the enhancer and the promoter. A spacer function of this kind might be particularly important in an LTR, like the triplication-containing LTR, in which the enhancer is not tandemly repeated. This possibility was discounted, however, by experiments in which two copies of the triplicated 21-bp sequence in the FeLV-945 LTR were replaced with 42-bp of random sequence. Modification of the LTR in this way was shown to greatly diminish the replicative capacity of the virus, although the spacing of regulatory elements within the LTR was maintained. We inferred from these findings that the 21-bp triplication does not perform solely a spacer function but that the sequence itself is important [16]. A second possibility was then considered, *i.e.*, that the 21-bp triplication contributes authentic enhancer function through the binding of nuclear transcription factors. In support of this possibility, it is noteworthy that the 42-bp contributed by two additional copies of the 21-bp repeat in the LTR represent an exact multiple of 10.5 bp per helical turn of DNA, *i.e.*, four whole turns [18,19]. Thus, nuclear protein binding sites encoded within the sequence would occur on the same face of the DNA molecule. Indeed, electrophoretic mobility shift assays (EMSA) using the 21-bp triplication as a probe demonstrated the formation of a specific protein-DNA complex using nuclear extracts prepared from cells in which the FeLV-945 LTR is preferentially active [16]. Sequence analysis indicated potential binding sites for the transcription factor, c-Myb, across the repeat junctions of the 21-bp triplication, a noteworthy finding in that such sites would not occur in the absence of the repeat; thus, a requirement for c-Myb binding to the repeat junctions of the triplication could represent the selective pressure to conserve its sequence precisely. Functional analysis demonstrated the specific binding of c-Myb to the triplication, and showed that the triplication-containing LTR is responsive to c-Myb in a manner that requires the presence of both c-Myb binding sites. Results further indicated that c-Myb in complex with the 21-bp triplication recruits the transcriptional co-activator, CREB-binding protein (CBP), to a DNA-protein complex assembled on the FeLV-945 LTR. In keeping with these results, FeLV-945 replication was shown to be positively responsive to CBP overexpression. Considering that CBP is present in hematopoietic cells in limiting amounts, and that the expression of CBP-responsive genes is regulated via competition for the limited availability of the transcriptional co-activator, we hypothesized that FeLV-945 replication in bone marrow may influence CBP availability and thereby alter the regulation of CBP-responsive genes known to be involved in normal hematopoiesis [20].

**Figure 4.**
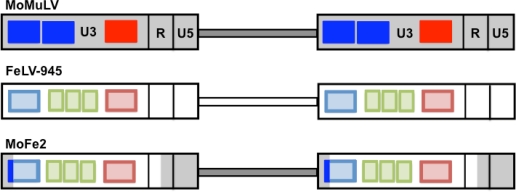
Schematic representation depicting the generation of the MoFe2-MuLV viral mutant. Represented are the proviral DNAs of Moloney murine leukemia virus (MoMuLV), FeLV-945, and the recombinant virus, MoFe2-MuLV (MoFe2). Indicated within the U3 region of each LTR are the number of enhancer repeats (blue boxes), the 21-bp sequence triplication (green boxes), and the transcriptional promoter (red box). The duplicated enhancer and promoter of MoMuLV were replaced by the corresponding sequence of FeLV-945 to generate the MoFe2 construct as shown.

While the study of FeLV pathogenesis *in vivo* in the natural host offers the potential for key mechanistic insights, manipulation of the cat model experimentally is hampered by several difficulties including the relative unpredictability of the outcome and the prolonged latency to malignant disease. With this in mind, we embarked on a study of the influence of the FeLV-945 LTR *in vivo* through the construction of a recombinant murine-feline retrovirus such that pathogenesis could be studied in the laboratory mouse. In collaboration with Dr. Hung Fan and colleagues, a recombinant virus, termed MoFe2-MuLV (MoFe2), was constructed in which the U3 region of the Moloney murine leukemia virus (Mo-MuLV) LTR was replaced with that of the triplication-containing FeLV-945 LTR (Figure 4). Mo-MuLV is a gammaretrovirus that uniformly induces T-cell lymphoma of the thymus with a relatively short latency of 3–4 months. The tumors induced by MoFe2 were exclusively thymic and of T-cell origin as evidenced by clonal, somatic rearrangement of the TCRβ locus in tumor DNA and a surface phenotype typical of immature and maturing thymocytes. These findings demonstrated that the presence of the FeLV-945 LTR was unable to re-direct disease spectrum of Mo-MuLV from T-cell lymphoma to non-T-cell disease associated with FeLV-945 in nature [21]. The results are consistent with studies demonstrating a replicative advantage conferred by the FeLV-945 LTR in feline T-cell lines [16], and with studies described below that implicate the FeLV-945 surface glycoprotein, but not the LTR, as the determinant of disease spectrum. As described below, the induction of T-cell lymphoma by MoFe2 proved to be a rich source for the identification of potential oncogenes, an indication that the unique MoFe2 LTR engaged a novel set of host genes in the induction of lymphoma.

## 3. Viral Surface Glycoprotein (SU) as a Determinant of Pathogenesis in the Cohort

**Figure 5.**
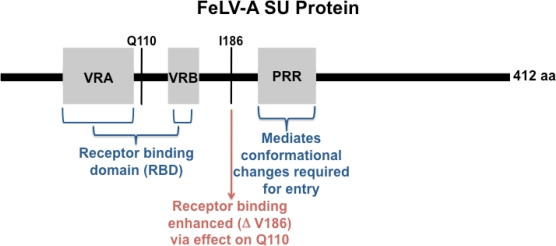
Diagrammatic representation of the FeLV-A surface glycoprotein (SU) protein. Shown is the 412 amino acid (aa) SU protein indicating approximate positions of the functional domains variable region A (VRA), VRB and proline-rich region (PRR). Mutation at the indicated residue I186 is implicated in determining receptor-binding phenotype via interaction with residue Q110.

Two proteins, the surface glycoprotein (SU) and the transmembrane protein (TM), are generated from the envelope gene of gammaretroviruses. SU resides on the particle surface, anchored to the TM protein, and is thereby positioned to make contact with the host cell surface and to interact directly with the receptor. The SU protein of gammaretroviruses contains three highly variable, functional domains required for receptor interaction and entry (Figure 5). Variable region A (VRA) is the primary determinant of receptor binding, and thus of host range, for FeLV and related retroviruses while variable region B (VRB) is a secondary determinant required for efficient infection [22–28]. An adjacent proline-rich region (PRR) mediates conformational changes required for virus entry [29]. These motifs comprise what has been termed the receptor binding domain (RBD). Outside of the conventionally defined RBD, additional binding determinants have been identified throughout the SU protein [24,30,31]. Entry of FeLV occurs after binding of the RBD to the host cell receptor, followed by conformational changes that ultimately lead to fusion of the viral and cellular envelopes. Thus, the SU protein acts as the initial determinant of tissue tropism, and the affinity of its interaction with host cell receptor may influence the rate of virus spread during infection.

Four subgroups of FeLV, namely FeLV-A, -B, -C, and -T, have been identified based on SU sequence and receptor utilization, and each subgroup has been associated with distinct pathogenesis [2,32–34]. Specifically, FeLV-A viruses represent the naturally occurring, horizontally transmissible subgroup spread cat-to-cat in nature. These viruses are weakly pathogenic, but can result in neoplastic disease, typically thymic lymphoma of T-cell origin, after a protracted asymptomatic phase. FeLV-B, C and T are thought not to be horizontally transmissible in nature, but arise *de novo* within the infected animal by mutation of FeLV-A and/or by recombination between FeLV-A and endogenous FeLV-related elements in the cat genome. The disease association of FeLV-B is not clearly understood, but FeLV-B infection is overrepresented in tissues from lymphoma relative to asymptomatic infected cats or other disease conditions. Infection with FeLV-C or FeLV-T is associated with anemia or immunodeficiency disease, respectively [1,2]. The receptors for each subgroup have been identified as multiple membrane-spanning proteins that function normally as transporters of small molecules. FeLV-A exhibits an ecotropic host range and utilizes a thiamine transporter, FeTHTR1, as receptor. FeLV-B and -C exhibit a broad host range, but utilize different receptors for entry. FeLV-B can use the phosphate transporters FePiT1 or FePiT2 as receptor, while FeLV-C uses a heme transporter, FLVCR. FeLV-T binds to FePiT1 as receptor, but is unique among the FeLVs because it requires a co-factor termed FELIX for entry into target cells [35,36]. Sequence analysis of SU proteins encoded by proviruses from diseased tissues in the cohort indicated that they were members of FeLV subgroup A, but that they were considerably more closely related to each other than to other FeLV-A SU proteins examined [5,37]. This finding was unexpected since the previously identified members of FeLV-A exhibit strong sequence conservation (∼97% amino acid sequence identity across SU) despite having been isolated over more than a decade from distant geographic locations across the world [2,3,38]. The largest sequence divergence between the cohort isolates and other FeLV-A SU proteins is located within the functional domains of the SU protein [37]. Specifically, the FeLV-945 SU protein was shown to be 89%, 73%, and 85% identical in predicted amino acid sequence to prototype FeLV-A/61E SU across VRA, VRB and PRR, respectively. In contrast, the segments of FeLV-945 SU between variable regions were 94% identical to FeLV-A/61E, and the 197 residues of the predicted TM product were 97% identical to FeLV-A/61E. In spite of the sequence differences, functional assays of receptor utilization including host range and superinfection interference confirmed the cohort viruses to be members of the FeLV-A subgroup [37]. Considering the assignment of cohort viruses to FeLV-A, the previous association of FeLV-B subtype viruses with T-cell lymphoma, and the predominance in the cohort of non-T-cell disease, a study was undertaken to determine to what extent viruses belonging to FeLV-B may contribute to the distinctive disease spectrum seen in the cohort [39]. The presence of FeLV-B in the genomic DNA of diseased tissues was examined by Southern blot analysis and PCR. FeLV-B was demonstrated in 50% of thymic lymphomas (2 of 4), a frequency comparable to that reported previously by us and others [40–44]. In contrast, FeLV-B was detected in only 25% of multicentric lymphomas, and was not detected in myeloproliferative disease or mast cell leukemia. These results confirm the association of FeLV-B with thymic lymphoma, but do not indicate a significant role in non-T-cell disease including multicentric lymphoma.

Having described the unique LTR and SU of FeLV-945, a study was undertaken to determine the role(s) of these sequence elements in determining the distinctive disease spectrum observed in the cohort, *i.e.*, a skew toward non-T-cell disease, particularly a multicentric lymphoma that excludes the thymus. For this purpose, recombinant viruses were generated in which either the FeLV-945 LTR alone or both the FeLV-945 LTR and SU gene were substituted for homologous sequences in FeLV-A/61E to generate the recombinant viruses termed 61E/945L or 61E/945SL, respectively. Experimental infection of neonatal kittens with 61E/945L recapitulated the pathogenesis observed with FeLV-A/61E, namely thymic lymphoma of T-cell origin, although disease occurred with significantly shorter latency. Thus, substitution of the FeLV-945 LTR into FeLV-A/61E did not alter the disease outcome but dramatically increased the pace of disease induction. In contrast, substitution of both the LTR and SU gene changed the disease outcome entirely, in that infection with 61E/945SL resulted in the rapid induction of multicentric lymphoma that involved lymphoid and non-lymphoid organs but excluded the thymus. Tumors induced by 61E/945SL were shown by flow cytometry and immunohistochemistry to be of B-cell origin. These findings identify the FeLV-945 LTR and SU gene as determinants of pathogenesis, indicating that the LTR determines the kinetics of disease induction and SU determines the tumorigenic spectrum [37,45]. Recently, an additional recombinant virus has been constructed in which only the SU gene of FeLV-945 was substituted into FeLV-A/61E to generate a recombinant virus termed 61E/945S. Preliminary findings indicate that infection of neonatal kittens with 61E/945S induces thymic lymphoma after prolonged latency, as does FeLV-A/61E. These findings support the role of the FeLV-945 LTR as the determinant of more rapid disease induction, and indicate that the FeLV-945 LTR and SU must act cooperatively to redirect tumorigenesis to the non-T-cell multicentric phenotype [46].

Considering that the FeLV-945 SU protein enters through the FeLV-A receptor, the possible mechanism by which it acts to redirect disease spectrum is not clear. Receptor-binding attributes of FeLV-945 SU were examined to explore whether a distinctive phenotype is apparent that may account for the unique pathogenesis of FeLV-945 [47]. Using flow cytometric binding assays, virions bearing the FeLV-945 envelope protein were observed to bind the cell surface receptor with significantly increased affinity, as was soluble FeLV-945 SU protein, when compared to the corresponding virions or soluble protein from FeLV-A/61E. This finding was confirmed in several feline cell lines as well as in mouse cells engineered to express FeTHTR1. Enhanced binding was observed over a 100-fold range of virus concentration, and thus, likely mimics *in vivo* conditions where amounts of virus or receptor may be limiting. We hypothesize that through its increased receptor binding affinity, FeLV-945 SU might affect pathogenesis and alter disease outcome by (i) increasing the rate of virus entry and spread *in vivo*, and/or (ii) facilitating entry into a novel target cell with a low receptor density. Studies were undertaken to identify which amino acid residues in FeLV-945 SU are responsible for the enhanced binding phenotype. Using the crystal structure of the RBD of FeLV-B as a model [48], computational molecular modeling was performed to compare the structures of the FeLV-A/61E and FeLV-945 SU proteins. The structure of the FeLV-A RBDs did not differ significantly; however, a prominent loop located within VRA was predicted from the models of FeLV-A/61E and FeLV-945 that is distinct from the known crystal structure of the FeLV-B RBD, and thus may represent the receptor-binding surface. Five residues were observed to differ between FeLV-A/61E and FeLV-945 within the loop, but reciprocal substitution of these residues did not alter the receptor-binding properties of the parent proteins. When larger substitution mutations were made covering the entire SU, only substitution of a region of FeLV-945 containing the VRB could confer enhanced binding to FeLV-A/61E SU. Mutational analysis of the sequence differences found in the VRB-containing region demonstrated that a single residue, valine 186 in FeLV-945 SU, could confer the enhanced receptor-binding phenotype when substituted into FeLV-A/61E. Of note is that the substitution, which replaces an isoleucine in FeLV-A/61E, is conservative and is located outside consensus VRB. Computational modeling predicts a mechanism by which the isoleucine-to-valine change at position 186 contributes to the binding phenotype of FeLV-945, *i.e.*, by its influence on a nearby conserved residue, glutamine 110 (Q110). The relatively bulky isoleucine side chain at position 186 effectively pushes Q110 into the lower end of a large binding cleft, thus narrowing the lower end. The more compact valine side chain, by contrast, is predicted to re-orient Q110 such that it does not protrude into the lower end of the binding cleft, thus widening the lower end and producing what may represent a better surface conformation for interaction with receptor. An inference of this model is that valine 186 acts to enhance the interaction of the receptor-binding domain, VRA, with its target [47].

## 4. Oncogene Activation in FeLV-Mediated Lymphomas in the Cohort

As a simple gammaretrovirus, the FeLV genome encodes the genes required for its structure and replication, *i.e.*, *gag*, *pol* and *env*, and no others; thus, unlike complex retroviruses or oncogene-containing retroviruses, FeLV encodes no gene to which its malignant potential can be directly attributed. It has long been recognized that FeLV, like other simple retroviruses, acts to induce neoplasia at least in part by activating cellular oncogenes at the sites of proviral integration. Thus, while the LTR acts to direct the expression of viral genes from the integrated provirus, it can act as well to promote or enhance transcription of adjacent gene sequences, including potential oncogenes. The consequent oncogene activation leads to cell transformation and expansion of a tumor in which the causal proviral integration is clonally represented. It is important to appreciate that the integration event near an oncogene is not thought to represent targeted integration; rather, it represents a selected event in that the cell thereby transformed gives rise to a clonal (or oligoclonal) tumor mass. For this reason, when the same genetic locus is observed to be interrupted by proviral integration in multiple independent tumors, it is inferred that the commonly interrupted locus encodes an oncogene whose activation is relevant to tumor induction [49–51]. Such a locus is then referred to as a common insertion site (CIS). FeLV is further known to interact with cellular oncogenes through a distinct mechanism, *i.e.*, retroviral transduction, in which the oncogene sequence is incorporated through a complex recombination event into the viral genome. Transmission of the oncogene-containing virus into a new target cell can then transform the cell directly. Analysis of the natural cohort of FeLV-infected animals described here has contributed to our understanding of both mechanisms of interaction and to the identification of new CISs and potential oncogenes (Table 1).

### 4.1. Oncogene Activation in Thymic Lymphomas

As demonstrated by studies from our laboratory and others over a period of years, FeLV-induced thymic lymphomas demonstrate a distinctive pattern of oncogene activation that involves proviral integration (or retroviral transduction) of *c-myc*, *bmi-1*, *pim-1* or *fit-1*, and frequently involves more than one of those loci in the same tumor [44,56]. Our early analysis of the thymic lymphoma of cat 1110 from the natural cohort demonstrated the unexpected finding of a novel provirus, designated LC-FeLV, that contained within it the full coding sequence of the *c-myc* oncogene. Presumably during infection in cat 1110, replicating FeLV had transduced *c-myc*, replacing the entire *pol* gene and part of the *gag* and *env* genes of the virus. The virus was thus rendered replication-defective but presumably oncogenic [52,57]. The oncogenic capacity of LC-FeLV was examined *in vitro*, by infection of early-passage feline leukocytes from peripheral blood, spleen, or thymus, or of neonatal feline fibroblasts. Unlike other oncogene-containing retroviruses, LC-FeLV did not immortalize these cells or alter them morphologically. In contrast, LC-FeLV infection of embryonic feline fibroblasts demonstrated partial transformation in that the cells were morphologically altered and demonstrated greatly increased proliferative potential, but failed to induce tumors when inoculated into athymic mice [58]. Analysis of the tumorigenic potential of LC-FeLV *in vivo* in cats similarly demonstrated an incomplete transforming potential. Specifically, neonatal animals were inoculated with LC-FeLV pseudotyped with replication-competent FeLV-A/Glasgow-1 and FeLV-B/Gardner-Arnstein. Of eight animals thus inoculated, only three developed thymic lymphoma but did so after a relatively short latency of 4–6 months. By comparison, littermates inoculated with helper viruses alone showed no signs of malignant disease at the time of necropsy after 11 months of infection [59].

The incomplete transforming potential of LC-FeLV *in vitro* in feline cells, together with the inefficient induction of thymic lymphoma in infected animals, suggested that the feline *v-myc* oncogene as represented in LC-FeLV is not sufficient to induce complete transformation, and that another genetic event(s) may be required. One possibility considered was that replicating FeLV in the infected animals may act as an insertional mutagen to disrupt a second oncogene, whose activation can then cooperate with feline *v-myc* to induce tumors. Analysis of this hypothesis using a strategy of transposon tagging demonstrated a locus, designated *flvi-2* (feline leukemia virus integration site-2), to be commonly interrupted by FeLV proviral insertion in six FeLV-induced thymic lymphomas including three induced by infection with LC-FeLV, thus identifying *flvi-2* as a CIS and a potential collaborator with feline *v-myc* in tumor induction [53]. The *flvi-2* locus was shown to encode *bmi-1* [60], a polycomb gene family member now known to play an essential role in embryogenesis, cell cycle regulation, and the control of self-renewal and differentiation of normal and leukemic stem cells [61,62]. The possible role of *bmi-1* as a *myc*-collaborator in the FeLV-mediated induction of lymphoma was further examined by screening a large collection of FeLV-positive feline lymphomas representing different cohorts and involving different strains of FeLV. In collaboration with several colleagues, the coincident involvement of *flvi-2* (*bmi-1*) and *myc* was examined in a broad geographic sampling of naturally and experimentally induced FeLV-positive lymphomas that were heterogeneous with respect to the strain of FeLV involved. The results demonstrated FeLV proviral insertions at the *flvi-2* locus in 7 of 18 thymic lymphomas examined (39%), four of which also exhibited alterations of the *myc* locus. Thus, consistent with our more limited earlier study [53], insertions at *flvi-2* were detected with approximately equal frequency in the presence and absence of *myc* involvement. These findings supported the concept that *bmi-1* can act as a *myc* collaborator, but that the interaction is not required for malignant induction; indeed, we hypothesized from these findings that proviral insertional activation of *flvi-2* may be an early event in a multistep oncogenic cascade, one possibility for completion of which is activation of *myc* [54]. A larger collaborative study confirmed and extended these findings by examining a series of 63 FeLV-positive T-cell lymphomas, including the thymic lymphomas from our natural cohort and those experimentally induced by infection with LC-FeLV. The tumors were examined for alterations in the *c-myc* and *flvi-2* loci, as well as in *fit-1* and *pim-1*, two other loci implicated as *myc*-collaborators in FeLV-induced disease [63]. The results demonstrated retroviral insertion or transduction at *c-myc*, *flvi-2*, *pim-1* or *fit-1* with varying frequencies as high as 32% in naturally occurring tumors. While interruptions of more than one of those loci within a single tumor was detected, the data clearly showed that each locus can contribute to disease induction independently, and that cooperative activation between them is not required [44].

### 4.2. Oncogene Activation in Multicentric, Non-T-Cell Tumors

In contrast to thymic lymphomas as described above, the multicentric, non-T-cell tumors in our collection demonstrated no involvement of *c-myc*, *flvi-2*, *fit-1* or *pim-1* loci as measured by Southern blot analysis for evidence of FeLV proviral insertion or transduction [5,44,54]. Rather, a locus in feline DNA termed *flvi-1* (feline leukemia virus integration site-1) was identified as a CIS in these tumors, shown to be interrupted by FeLV proviral integration in 4 of 11 multicentric lymphomas [5,14]. While the coding capacity of *flvi-1* remains unknown, the locus is phylogenetically conserved among mammals and has been localized to mouse chromosome 2E, adjacent to the known oncogene *spi-1* [64].

Considering its evolutionary conservation and implication as a CIS, *flvi-1* is thought to encode an as yet unrecognized oncogene whose activity is affected by the nearby integration of the unique FeLV-945 LTR. To further explore potential oncogenes whose activity may be altered by adjacent integration of the unique LTR, we turned to the large set of lymphomas induced by MoFe2, the recombinant virus described above in which the U3 region of the FeLV-945 LTR was substituted for that of MoMuLV. While the tumors induced by MoFe2 infection were uniformly thymic lymphomas of T-cell origin, they differed from those induced by either parent virus with respect to the pattern of oncogene involvement. Specifically, among 44 tumors examined, MoFe2 integration was found infrequently (0%–9%) near CISs previously identified for either MoMuLV or FeLV-945 [21,55]. Thus, a hunt for new CISs ensued using three different approaches, the results of which identified six loci as targets for common MoFe2 insertion. Of these, the loci encoding the genes *Rasgrp1*, *Jundm2*, *Ahi-1*, and *Rras2* had been previously identified as a CIS in other retroviral models. A CIS was identified for the first time that encodes the p101 regulatory subunit of phosphoinositide-3-kinase gamma (PI3Kγ), a key regulator of T-lymphocyte proliferation and cytokine production. Identification of the p101 gene as a CIS in MoFe2-induced tumors suggested that it may act as an oncogene in the induction of T-cell lymphoma in this model. To explore this possibility further, we examined the effects of p101 expression and PI3Kγ signaling on T-cell growth and survival. The results provided the first evidence that p101 overexpression alone can activate the PI3Kγ pathway by activating the catalytic subunit, p110γ, and by sensitizing it to activating signals. Further analysis in human T-cell lines demonstrated that moderate levels (but not high levels) of p101 overexpression protected cells from apoptosis through a mechanism apparently mediated by Akt phosphorylation [65]. Of the six CISs identified in MoFe2-induced tumors, none had previously been reported in tumors induced by either parent virus in wild type animals, and two had not previously been reported in any model. Thus, substitution of FeLV-945 LTR sequences into MuLV significantly altered the pattern of oncogene utilization. These findings indicate that the distinctive sequence and/or structure of the FeLV-945 LTR determines its pattern of insertional activation, and demonstrate its utility as a tool for the identification of new oncogenes [55,65].

## 5. Conclusions

FeLV-induced lymphoma is a multistep process that involves complex and interacting genetic determinants encoded both by the virus and the host. A collection of diseased tissues from a temporal and geographic cohort of naturally infected cats has been extensively examined to identify determinants of thymic or multicentric lymphoma in the natural host. The results revealed predominant but distinct virus isolates associated with lymphomas of each type. The findings were applied to experimental systems, including infections of the natural host with recombinant viruses bearing the distinctive features of predominant isolates, and the development of novel MuLV-FeLV recombinant viruses such that pathogenesis could be studied in the laboratory mouse. The results of these and other studies implicated as major disease determinants the unique FeLV LTR and SU present in the predominant natural isolate, and further identified distinct sets of oncogenes activated in each type of lymphoma. These studies demonstrate the value and utility of natural clinical material in understanding the selective pressures operative in infection of the natural host and the complex interplay of viral and host factors that give rise to disease.

## Figures and Tables

**Table 1.** Common Integration Sites (CIS) for FeLV as studied through analysis of the natural cohort.^a^

**CIS**	**Virus**	**Tumor Type^b^**	**Gene Function**	**Reference**
*c-Myc*	FeLV	Thymic lymphoma	Controls cell proliferation and survival	[44,52]
*flvi-2* = *bmi-1*	FeLV, LC-FeLV	Thymic lymphoma	Controls self-renewal *vs.* differentiation in hematopoiesis	[44,53,54]
*pim-1*	FeLV	Thymic lymphoma	serine-threonine kinase; survival signaling	[44]
*fit-1*	FeLV	Thymic lymphoma	Unknown	[44]
*flvi-1*	FeLV-945	Multicentric lymphoma	Unknown	[5,14]
*p101*	MoFe2	Thymic lymphoma	Regulatory subunit of PI3Kγ	[55]
*Rasgrp1*	MoFe2	Thymic lymphoma	Ras guanyl releasing protein; cell signaling	[55]
*Jundm2*	MoFe2	Thymic lymphoma	Jun dimerization protein (predicted); cell signaling	[55]
*Ahi-1*	MoFe2	Thymic lymphoma	Abelson helper virus integration site; associated with neurologic and hematologic disorders	[55]
*Rras2*	MoFe2	Thymic lymphoma	Ras-related protein; cell signaling	[55]

a Table 1 summarizes common integration sites studied through analysis of the natural cohort and/or through experimental infection with viruses derived from the cohort. Table 1 is not intended to represent an inclusive summary of all identified common integration sites for FeLV.

b Indicated is the tissue in which the common integration was observed.
